# Mortality Impact of Severe COVID-19 in the ICU: A Study from the Târgu Mureș Support Unit

**DOI:** 10.3390/life14101232

**Published:** 2024-09-26

**Authors:** Janos Szederjesi, Irina Săplăcan, Marius Petrișor, Alexandra-Maria Șerdean, Bianca-Liana Grigorescu

**Affiliations:** 1 Department of Intensive Care, George Emil Palade University of Medicine, Pharmacy, Science and Technology, 540139 Târgu-Mureș, Romania; yangzi37@gmail.com (J.S.); serdean.alexandramaria@gmail.com (A.-M.Ș.); biancagrigorescu20@yahoo.com (B.-L.G.); 2 Department of Simulation Applied in Medicine, University of Medicine, Pharmacology, Science and Technology, 540139 Târgu-Mureș, Romania; mariuspetrisor@gmail.com

**Keywords:** COVID19, SARS-CoV-2, mortality

## Abstract

(1) Background: Since the onset of the COVID-19 pandemic, it has been recognized that a considerable proportion of critically ill patients may die of this disease. The current study aims to assess the overall 1-year outcomes within the UMFST COVID-19 Unit, providing valuable insights into the efficacy of specialized care facilities in managing severe cases of COVID-19. (2) Methods: This is a retrospective monocentric observational study including 294 patients confirmed to have SARS-CoV-2 infection. Demographic data and clinical and paraclinical parameters were assessed. Survival probabilities were estimated using Kaplan–Meier curves. (3) Results: Overall, the 1-year mortality was 89.4%. All deaths occurred in-hospital, with two patients dying after 28 days. Diabetes mellitus, chronic kidney failure, cerebrovascular disease, and atrial fibrillation were more prevalent in deceased patients. Thirty percent of patients needed endotracheal intubation during the first 24 h. The incidence of hospital-acquired pneumonia was higher among deceased patients. The SOFA score was significantly different between deceased vs. survivors. The survival analysis showed that the use of noradrenaline increased the likelihood of surviving COVID-19. (4) Conclusions: The severe comorbidities of the patients were the primary factors contributing to the increased mortality rate in the COVID-19 unit.

## 1. Introduction

Despite years of research regarding the pathophysiology and management of Acute Respiratory Distress Syndrome (ARDS), the pandemic of SARS-CoV-2 infection represented a deadly race for worldwide intensivists. On 11 February 2020, the World Health Organization (WHO) formally designated the illness caused by SARS-CoV-2 “coronavirus disease 2019” (COVID-19). COVID-19 encompasses a range of clinical symptoms, usually consisting of a fever, dry cough, and fatigue, frequently accompanied by lung involvement [1].

ARDS is a severe and potentially deadly complication of COVID-19, with a death rate ranging from 23% to 56% [2,3]. The high mortality attributable to SARS-CoV2 infection can be considered a consequence of direct respiratory impairment as well as a life-threatening result of an enormous tissular oxygen demand, leading to mitochondrial dysfunction and multiple organ failure. Since the onset of the COVID-19 pandemic, it has been recognized that a considerable proportion (up to one-third) of critically ill patients who are hospitalized may die of this disease. To date, more than 773 million people worldwide have been infected, and almost 7 million have died of COVID-19. In Romania, more than 3.5 million confirmed cases of COVID-19 and over 68,000 deaths have been recorded [4].

Due to the extent, severity, and rapid onset of symptoms, with potentially lethal progression, the COVID-19 pandemic imposed a significant financial burden on both patients and the public. Aside from financial resources, it necessitated the availability of human and medical resources [5]. The surge in critically ill patients, coupled with insufficient bed capacity to accommodate the increasing demand, necessitated the establishment of dedicated COVID-19 support units worldwide. Given the unprecedented challenges posed by the COVID-19 pandemic, there is an urgent need to assess the effectiveness of specialized care units such as the UMFST COVID-19 Support Unit. Understanding the high mortality rates within these units is vital for gauging their impact on patient outcomes and guiding future resource allocation and healthcare strategies. In September 2020, the UMFST COVID-19 Support Unit was established in Târgu Mureș, Romania, in the Multipurpose Hall of the George Emil Palade University of Medicine, Pharmacy, Science, and Technology, with a capacity of 40 critical care beds, as an external ward of the Emergency County Clinical Hospital from Targu-Mures. This facility treated patients from all regions of the country, the majority of whom were beyond the reach of medical resources. The Emergency County Clinical Hospital in Târgu Mures also set up an ICU (intensive care unit) designed to accommodate COVID-19-positive patients with fewer comorbidities and a higher likelihood of survival during the pandemic.

The present study aimed to evaluate the overall 1-year management and outcomes within the UMFST COVID-19 Unit, providing valuable insights into the efficacy of specialized care facilities in managing severe cases of COVID-19.

## 2. Materials and Methods

We conducted a retrospective monocentric observational study encompassing 294 adult patients confirmed to have SARS-CoV-2 infection and hospitalized in the COVID-19 UMFST Support Unit of the Emergency Clinical County Hospital, Târgu Mures, Romania, between 1 January 2021 and 31 December 2021. The inclusion criteria for the COVID-19 group were an age of between 18 and 90 years and ICU admission for COVID-19. Nasopharyngeal swab samples were obtained from the patients, and a COVID-19 diagnosis was established using real-time reverse transcription polymerase chain reaction (RT–PCR) analysis.

Demographic data were obtained for all patients. Several clinical and paraclinical parameters were assessed: serial bacteriological tests, blood counts, biochemical tests, and serum lactate, blood glucose, albumin, total protein, and arterial blood gas analyses. The Acute Physiology and Chronic Health Evaluation score (APACHE II) and Sequential Organ Failure Assessment score (SOFA) were calculated for every patient for each day. The mode of mechanical ventilation and ventilatory parameters were recorded, as was the vasoactive/inotrope medication administered.

Patients were treated according to the international recommendations for COVID-19 infection, which included routine thromboprophylaxis depending on the risk of bleeding, as well as antivirals, corticosteroids, or the IL-6 (Interleukine-6) receptor antagonist tocilizumab (Roche Registration GmbH, Grenzach-Wyhlen, Germany). At the time of the study, the antivirals used included favipiravir (Meditop Gyógyszeripari, Pilisborosjenő, Hungary) and remdesivir (Gilead Sciences, Carrigtwohill, Ireland). Corticosteroid therapy consisted of dexamethasone or methylprednisolone administered for at least 10 days. Antibiotics were used to treat bacterial co-infections and immunocompromised patients according to antibiograms.

This study was approved by the local Hospitals Ethics Committee (approval no 7390/15 March 2023). The General Data Protection Regulation (GDPR) agreement was respected, and the obtained data were used for research purposes only. During the first peak of the pandemic in Romania, a written agreement was not obtained to prevent paper contamination, as per the hospital regulations at the time.

### Statistical Analysis

The obtained data were recorded in a database and statistically analyzed using SPSS Statistics 17.0. Data series normality was tested using the Kolmogorov–Smirnov test. No normal distributions were identified among the analyzed variables. Descriptive statistics are reported as median, minimum, maximum, percentiles (25th and 75th), and interquartile range (IQR).

Survival probabilities were estimated using Kaplan–Meier curves, and the significance of variations in survival curves was assessed using the log-rank test. The survival odds were reported on the final day of either discharge or death.

The survival analysis was expanded by using the Cox proportional hazards model to estimate the probability of death and simultaneously evaluate the impact of many risk factors on survival while accounting for confounding or effect modification. The use of inotropes/vasoactive drugs and bacterial superinfection were used as variables in the Cox proportional hazards model.

## 3. Results

A total of 294 COVID-19 patients were hospitalized at the UMFST COVID-19 Support Unit affiliate of the Emergency Clinical County Hospital (Targu Mures, Romania), with laboratory-confirmed SARS-CoV-2 infection, between January and December 2021. Overall, the 1-year mortality was 89.4% (*n* = 262/294). All deaths occurred in-hospital, with two patients dying after 28 days (5.24% of the overall in-hospital mortality, *n* = 2/262); see Table 1.

During the first 24 h following hospital admission, all patients required oxygen or ventilatory support. Table 2 and Table 3 show the severity of respiratory failure, as well as the cardiac and respiratory rates, gas exchange, and ventilation support. Continuous positive airway pressure (CPAP) or noninvasive mechanical ventilation (NIV) was required in 57.33% (*n* = 168) of the cases, and 30% (*n* = 86) of patients needed endotracheal intubation (ETI) during the first 24 h.

APACHE II and SOFA scores were calculated for each patient on the first day of admission. The length of stay (LOS) in days in the ICU and severity scores are shown in Table 4.

Throughout the study, patients were treated for COVID-19 using antiviral drugs including remdesivir (*n* = 171), favipiravir (*n* = 64), and the monoclonal antibody tocilizumab (*n* = 25), all of which were prescribed in accordance with the established protocol. Table 5 provides details regarding the administration of remdesivir, favipiravir, and tocilizumab.

During hospitalization, 37.74% (*n* = 93) of patients had positive cultures (Table 6).

**Figure 1:**
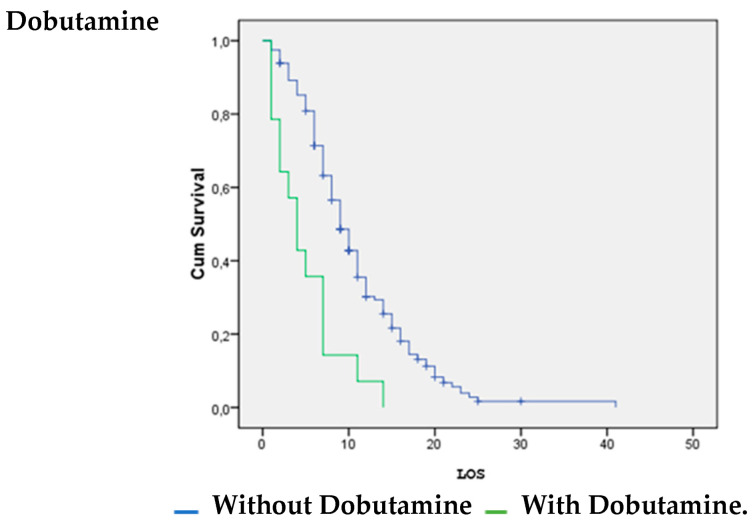
Dobutamine vs. LOS.

**Figure 2:**
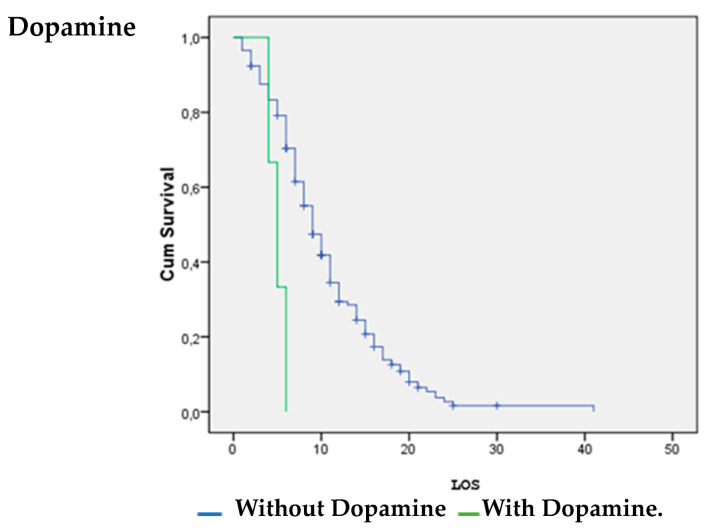
Dopamine vs. LOS.

**Figure 3:**
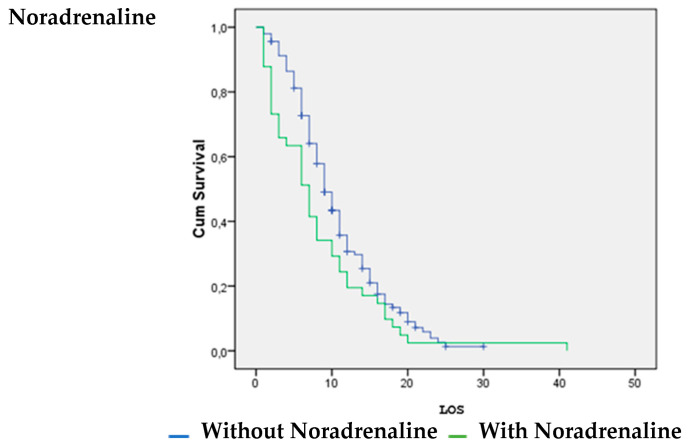
Noradrenaline vs. LOS.

Patients with vasoactive support with noradrenaline were significantly more likely to survive (*p* = 0.026). Patients without inotropic support with dobutamine (*p* = 0.00) and dopamine (*p* = 0.008) were significantly more likely to survive (Figure 1, Figure 2 and Figure 3).

**Figure 4:**
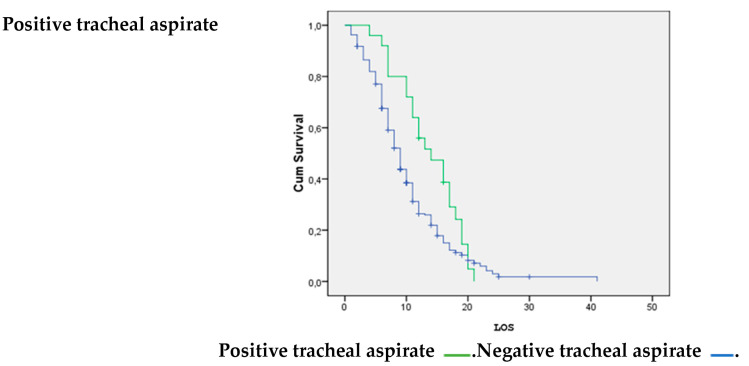
Positive tracheal aspirate vs. LOS.

Patients without a positive tracheal aspirate were significantly more likely to survive than patients with bacterial superinfection (*p* = 0.036); see Figure 4.

## 4. Discussion

The exceptionally high 1-year mortality rate of 89.4% among COVID-19 patients admitted to the UMFST COVID-19 Support Unit in Targu Mures, Romania, underscores the severe impact of the disease on patient outcomes. This finding is consistent with reports from various healthcare settings globally, highlighting the substantial morbidity and mortality associated with COVID-19, particularly among critically ill patients requiring hospitalization [6,7]. The mortality rates in intensive care units associated with severe acute respiratory syndrome coronavirus 2 (SARS-CoV-2) exhibited variation throughout the course of the pandemic on a global scale. In 2023, Chandel A. and colleagues conducted a meta-analysis examining mortality rates among patients with COVID-19 who required hospitalization, ICU admission, and organ support. The recorded ICU mortality rates in the 42 countries included in the study ranged from 15% to 84% [8]. The fact that all deaths occurred in-hospital further emphasizes the gravity of illness experienced by these patients and the challenges faced by healthcare providers in managing severe cases of COVID-19.

The intensive care unit at Emergency Clinical County Hospital, Târgu Mures, Romania, has been categorized into separate sections for “COVID-19 and non-COVID-19” patients. In response to the surge in infected patients presented in a critical condition, alongside the necessity to accommodate non-COVID cases, including complex polytrauma, neurosurgery, and general surgery, the COVID-19 support unit was established within the multipurpose room of the University of Medicine, Pharmacy, Sciences and Technology of Târgu Mureş. Patients with diminished survival chances, as well as those transferred from various regions of the country due to insufficient ICU availability, were admitted to this unit.

The advent of new variants of SARS-CoV-2 underscores a significant problem of this epidemic. The mutations resulting from successive viral replication were a normal occurrence. The SARS-CoV-2 virus evolves at a pace of 1.1 × 10 − 3 substitutions per site annually [9].

B1.1.7, commonly referred to as the alpha variant, was among the initial notable variants, exhibiting 23 mutations, and raised significant alarm in September 2020 because of its 56% increased transmissibility. A more aggressive version, B.1.351, or the beta variant, surfaced in South Africa in October 2020 and was found to be 50% more transmissible. People affected by the alpha variant demonstrated no immunity to the South African variant [9,10].

In 2021, the delta variant became associated with a significant increase in transmissibility, exhibiting a 60% rise in hospitalization rates. Additionally, it presents higher viral loads and a diminished response to existing vaccines, which are critical factors contributing to its classification as a variant of concern [11]. Nytia Kumar et al. conducted a study involving approximately 1400 patients and found that patients infected with the delta variant experienced a prolonged recovery period. This was indicated by a longer LOS and increased viral shedding in comparison to those infected with the alpha variant. This pattern was consistently observed in both vaccinated and unvaccinated patients [12]. The patients participating in the present study were predominantly infected with the beta, gamma, or delta variants, which were responsible for the highest number of infections in 2021.

The clinical outcomes and LOS are directly related to the underlying health conditions and age of the patient with COVID-19. In numerous publications, cardiometabolic syndrome, encompassing obesity, type 2 diabetes, and hypertension, emerged as the principal comorbidity correlated with elevated mortality rates [13,14,15]. Priya Singh et al. conducted a study in India that concluded that major risk factors for mortality were associated with diabetes and hypertension. An elevated risk of mortality was similarly linked to cardiovascular disease and chronic lung diseases [16].

In our study, most patients, regardless of their survival status, had a BMI below 30. Morbid obesity, defined as a BMI greater than 40, was observed more frequently among deceased patients, with a prevalence of 2.7% compared to 0.7% in those who survived. Hypertension was the predominant comorbidity identified in both the deceased (N = 188, 71.7%) and surviving (N = 17, 5.87%) patients. Chronic heart failure was noted with greater prevalence in patients who did not survive (N = 118, 40%) in contrast to those who did (N = 5, 1.7%). Diabetes mellitus was noted solely in deceased patients, representing 30.27% of the cases. Among the deceased patients, 64% were diagnosed with chronic heart disease, whereas only 1.7% of those who survived had this condition. Cerebrovascular disease was observed in 10% of deceased patients compared to 0.3% in survivors. Both groups presented with conditions including COPD, pulmonary fibrosis, and hepatic cirrhosis, with a notably higher prevalence observed among the deceased patients. Yousof Khairy and colleagues conducted a systematic review involving over 1.2 million patients, revealing a prevalence of hypertension of 25%. This condition was identified as a major contributor to mortality, prolonged hospitalization, and an elevated risk of ICU admission [17]. Vardavas C. and colleagues conducted a meta-analysis revealing a strong relationship between certain prognostic factors, including diabetes insipidus, heart failure, stroke, diabetes, and end-stage renal disease, and in-hospital mortality [18]. In our study, diabetes mellitus, atrial fibrillation, previous myocardial infarction, and a history of cancer were noted solely in the deceased group

The median PaO_2_ levels were notably lower in the deceased group than in survivors. Hypoxemia is a common feature of severe COVID-19 and is associated with adverse outcomes such as respiratory failure and mortality [19]. A lower PaO_2_/FiO_2_ ratio is indicative of impaired gas exchange and respiratory failure. The significantly lower median ratio in the deceased group suggests more severe respiratory compromise and a poorer prognosis compared to survivors. Moreover, we found that the RI was significantly higher in the non-survivors’ group (*p* = 0.009), which indicated a profound and severe alteration in gas exchange at the alveolo-capillary membrane and can be considered an indirect parameter for the severity of ARDS.

The oxygen requirements in our study indicate a substantial difference in the utilization of noninvasive mechanical ventilation between survivors and deceased patients. A significantly higher proportion of deceased patients required NIV compared to survivors in the first 24 h after admission to the ICU. The need for ETI and invasive mechanical ventilation was notably higher among deceased patients than among survivors. This finding underscores the severity of respiratory failure and the need for aggressive airway management in critically ill COVID-19 patients. Overall, the data suggest that non-survivors in this study cohort exhibited a greater need for advanced respiratory support in the first 24 h of admission, as evidenced by higher rates of NIV and ETI utilization. In contrast, survivors may have been managed with less invasive respiratory support. Our results are consistent with those from other studies. We could identify consistent patterns and trends in the utilization of NIV among COVID-19 patients with different clinical outcomes. Sara C. Auld et al. found a higher mortality rate in their study of intubated patients [20]. Sara Manrique et al. found that early intubation within the first 24 h of ICU admission in patients with COVID-19-associated pneumonia was an independent protective factor against mortality. Additionally, their data emphasized the critical role of invasive mechanical ventilation in managing severe respiratory failure associated with COVID-19 [21]. Furthermore, most patients admitted to the support unit did not fulfill the criteria for tracheostomy, and patients who were eligible were in a critical state that precluded their transfer to the County Hospital, where appropriate operating rooms for performing the tracheostomy were available.

According to the mean SOFA score of admission days and the APACHE II score, the ICU death rate in this clinical observational study was 14.41 points, with a mean estimated mortality of 13.43% and 6.17 points, respectively. However, the actual death rate (89.4%) differed significantly from the SOFA and APACHE II mortality predictions. In a study of 292 patients from multiple centers, Muhammad Monk et al. found that the SOFA score was the best predictor, even though there were no statistically significant correlations between the SOFA score and mortality [22].

In our study, the APACHE II scores indicated significant variability in the severity of illness among both survivors and deceased patients. While the median APACHE II scores were similar between survivors and deceased patients, the maximum scores were notably higher in deceased patients but without being statistically significant (*p* = 0.065). In the case of the SOFA score, we obtained a statistically significant difference between the deceased and the survivors (*p* = 0.001). This suggests that although the overall severity of illness at admission may have been comparable, there were patients with extremely severe illness who did not survive due to their comorbidity’s complications.

The LOS in the hospital for COVID-19 could help determine the disease’s prognosis. In a critical circumstance, like the COVID-19 pandemic, estimating the LOS for ICU patients is essential for allocating financial and human resources, as well as available beds [23]. Babak Jamshidi studied the lengths of hospitalization for fatal cases in the United States, Italy, and Germany, which were 2–10, 1–6, and 5–19 days, respectively [24]. In our study, the median LOS for the entire group was 9 days, with a mode of 6 days. In the case of deceased patients, the median was lower than in the survivor group (9.54 vs. 11.66 days). In the entire patient group, two patients exceeded 25 days of ICU stay, both non-survivors.

The trial conducted by the WHO Solidarity Trial Consortium in 2020 indicates that remdesivir, hydroxychloroquine, lopinavir, and interferon regimens exhibit minimal or potentially negligible effects on the mortality rates of patients with COVID-19 [25].

In our study, remdesivir (200 mg/day on day 1, then 100 mg/day for 5–10 days) and favipiravir (1600 mg every 12 h on the first day, then 600 mg every 12 h for 10–14 days) as antiviral treatments and immunomodulatory treatment with tocilizumab 8 mg/kg (maximum 800 mg, 1–2 administrations) and dexamethasone 6 mg/day were administered to the COVID-19 group according to national protocols at the time of data collection and the recommendation of the infectious disease physician. We did not notice a significant difference between survivors and non-survivors in our study groups, related to the specific antiviral treatment.

The survival analysis and estimation of the hazard ratio of death showed that the use of noradrenaline increased the likelihood of surviving COVID-19. However, the use of dobutamine and dopamine increased the likelihood of death. A systematic review by Maria Mermiri et al. determined that the utilization of vasopressors leads to an elevated in-hospital mortality rate and is linked to an increased risk of developing acute renal injury [26].

Bacterial superinfection in patients with critical COVID-19 can be caused by various factors and can be considered an additional factor increasing mortality. Some notable factors include the administration of high doses of corticosteroids for an extended period, oro-tracheal intubation, and a prolonged stay or previous pulmonary disease such as COPD or pulmonary fibrosis [27]. The survival analysis performed on pulmonary infections demonstrated that a positive tracheal aspirate could significantly increase the probability of mortality.

The increased mortality noted in the external ward for COVID-19 can be attributed to various comorbid conditions, the high rate of bacterial superinfections, and logistic difficulties like challenges in finding an available intensive care bed and the necessity of transporting these patients from various parts of the country. Many patients presented in a critical state, having been admitted to medical wards despite meeting criteria for intensive care. This situation often arose due to a lack of available beds or patients opting to remain at home until their condition worsened, ultimately necessitating invasive ventilatory support.

## 5. Conclusions

The severe comorbidities of the patients were the primary factors contributing to the increased mortality rate (89%) in the COVID-19 unit. Multiple comorbidities and cardiovascular sequelae may explain this observation. The incidence of hospital-acquired pneumonia was higher among patients who died in the hospital. Additionally, patients from nearly every region of Romania were relocated to this facility, as they were deemed to need further therapeutic resources and no longer had a bed available in their hometown. This study illustrates the initial waves of COVID-19 and the formidable challenges faced throughout the pandemic crisis.

The COVID-19 pandemic imposed a significant strain on both patients and hospitals as a result of the rapid onset, severity, and extent of symptoms. The widespread skepticism within the general population towards the healthcare system has led to patients frequently pursuing hospital care only after considerable progression of pulmonary damage, which subsequently limits the treatment options that can be offered.

The primary limitation of this single-center study is the inclusion of patients from the initial wave of the pandemic when the healthcare system was overburdened. As we studied a support unit, most patients were admitted in a critical condition, “ante finem”. Furthermore, there was a notable turnover of patients within a restricted number of beds, with the average length of stay recorded at 9.45 days, varying from 1 to 41 days. The participants were enrolled in the study sequentially, without strict inclusion criteria.

## Figures and Tables

**Table 1:** Demographic data and comorbidities.

All Patients (*n* = 294)	Overall Mortality	
	Alive (*n* = 32)	Deceased (*n* = 262)
Female gender	13	124
Male gender	18	138
Age, median (IQR)	61	69
BMI < 30	24	172
BMI ≥ 30	6	82
Severe obesity	2	8
Hypertension	17	188
Diabetes mellitus	0	89
Chronic kidney failure	3	34
Chronic obstructive pulmonary disease (COPD)	1	22
Pulmonary fibrosis	1	10
Active hematological malignancy	0	3
Cancer history	0	7
Cerebrovascular disease	1	29
Previous myocardial infarction	0	19
Atrial fibrillation	0	42
Chronic heart failure	5	118
Hepatic cirrhosis	7	1

**Table 2:** Arterial blood gas analysis.

	Alive (*n* = 32)	Deceased (*n* = 262)	
	Median (Minimum–Maximum)		*p*-Value
pH	7.46 (7.27–7.56)	7.42 (6.87–7.62)	0.0030 *
PaO_2_ (mmHg)	82.85 (55.8–226.3)	74.5 (24.3–385)	0.0999
PaCO_2_ (mmHg)	31.8 (19.2–61.9)	32.9 (12.6–104)	0.6107
PaO_2_/FiO_2_	169 (55.8–441.8)	86.6 (36–514)	0.0011 *
HCO_3_ (mmol/L)	24.55 (11.8–35.8	20.9 (6–58.8)	0.0263
FiO_2_ (%)	63 (40–100)	100 (21–100)	<0.0001
SaO_2_(%)	96 (76–99)	93 (53–100)	0.1553
RI	3.9 (0.3–10)	6.25 (0–17.4)	0.0092
Lac (mmol/L)	1.3 (0.6–4.2)	1.7 (0.4–15)	0.0693

* Man–Whitney. Arterial pressure of oxygen (PaO_2_); ratio of partial pressure of oxygen in arterial blood to the inspiratory oxygen concentration fraction (PaO_2_/FiO_2_); arterial bicarbonate (HCO_3_); fraction of inspiratory oxygen concentration (FiO_2_); arterial pressure of carbon dioxide (PaCO_2_); arterial oxygen saturation (SaO_2_); respiratory index (RI); lactate (Lac).

**Table 3:** Oxygen requirement in the first 24 h.

	Alive (*n* = 32)	Deceased (*n* = 262)
FM	16 (50%)	20 (7.6%)
HF	3 (9.37%)	1 (0.3%)
NIV	10 (31.25%)	158 (60.3%)
ETI	3 (9.3%)	83 (31.6%)
FM	16 (50%)	20 (7.6%)

Facial mask (FM); high flow (HF); noninvasive mechanical ventilation (NIV); endotracheal intubation (ETI).

**Table 4:** APACHE II, SOFA scores on day 1 of ICU admission, and LOS.

	All Patients (N = 294)	Alive (*n* = 32)	Deceased (*n* = 262)	*p* Value
APACHE II (points)				
Minimum	1	3	1	
Maximum	47	33	47	0.065
Mean	14.18	12.23	14.41	
50th (median)	13	13	13	
APACHE mortality (%)				
Minimum	1	1	1	
Maximum	87	71	87	0.157
Mean	13.43	9.8	13.89	
50th (median)	6	11	6	
SOFA (points)				
Minimum	1	2	1	
Maximum	24	9	24	0.001
Mean	6.17	3.68	6.44	
50th (median)	5	3	5	
LOS				
Minimum	1	2	1	
Maximum	41	30	41	0.067
Mean	9.75	11.66	9.54	
50th (median)	9	10	8.5	

**Table 5:** COVID-19-specific treatment.

	Number of Patients N (%)	Alive N (%)	Deceased N (%)
Remdesivir	193 (65.87%)	22 (11.4%)	171 (88.6%)
Favipiravir	64 (21.84%)	8 (12.5%)	56 (87.5%)
Tocilizumab	25 (8.5%)	3 (12%)	22 (88%)

**Table 6:** Superinfections.

Sample	Alive (*n* = 32)	Deceased (*n* = 262)
Blood cultures	1	19
Transtracheal aspiration	2	27
Clostridium difficile infection	2	5
Urinary culture	0	7
Infected sores	2	26

## Data Availability

The data used for this study can be found in the database of the Târgu Mures County Emergency Clinical Hospital, Romania.
